# A time fractional model of a Maxwell nanofluid through a channel flow with applications in grease

**DOI:** 10.1038/s41598-023-31567-y

**Published:** 2023-03-17

**Authors:** Naveed Khan, Farhad Ali, Zubair Ahmad, Saqib Murtaza, Abdul Hamid Ganie, Ilyas Khan, Sayed M. Eldin

**Affiliations:** 1 Department of Mathematics, City University of Science and Information Technology, Peshawar, 25000 Khyber Pakhtunkhwa Pakistan; 2 Basic Sciences Department, College of Science and Theoretical Studies, Saudi Electronic University, Abha Male, 61421 Saudi Arabia; 3 Department of Mathematics, College of Science Al-Zulfi, Majmaah University, Al-Majmaah, 11952 Saudi Arabia; 4 Center of Research, Faculty of Engineering, Future University in Egypt, New Cairo, 11835 Egypt

**Keywords:** Mechanical engineering, Applied mathematics, Nanoscience and technology

## Abstract

Several scientists are interested in recent developments in nanotechnology and nanoscience. Grease is an essential component of many machines and engines because it helps keep them cool by reducing friction between their various elements. In sealed life applications including centralized lubrication systems, electrical motors, bearings, logging and mining machinery, truck wheel hubs, construction, landscaping, and gearboxes, greases are also utilized. Nanoparticles are added to convectional grease to improve its cooling and lubricating properties. More specifically, the current study goal is to investigate open channel flow while taking grease into account as a Maxwell fluid with *MoS*_*2*_ nanoparticles suspended in it. The Caputo-Fabrizio time-fractional derivative is used to convert the issue from a linked classical order PDE to a local fractional model. To determine the precise solutions for the velocity, temperature, and concentration distributions, two integral transform techniques the finite Fourier sine and the Laplace transform technique are jointly utilized. The resultant answers are physically explored and displayed using various graphs. It is important to note that the fractional model, which offers a variety of integral curves, more accurately depicts the flow behavior than the classical model. Skin friction, the Nusselt number, and the Sherwood number are engineering-related numbers that are quantitatively determined and displayed in tabular form. It is determined that adding *MoS*_*2*_ nanoparticles to grease causes a 19.1146% increase in heat transmission and a 2.5122% decrease in mass transfer. The results obtained in this work are compared with published literature for the accuracy purpose.

## Introduction

Both Newtonian and non-Newtonian fluids are prevalent in nature. Simple Newtonian fluids did not adequately explain many difficulties in nature in the beginning. Numerous researchers have offered various non-Newtonian models that are not adequately covered by the straightforward Navier–Stokes theory in order to investigate these issues. Exact solutions to problems involving the free convection flow of viscous fluid are widely available in the literature. Because they are so common, non-Newtonian fluids are of interest to researchers. Researchers have proposed a number of mathematical models to comprehend the mechanics of non-Newtonian fluids since they have a wide variety of physical structures. These models are categorized as rate-type fluids or general differential form fluids. Maxwell^1^ presents the Maxwell fluid idea.

Maxwell nanofluid flow across a porous rotating disk with the effect of heat transfer was studied by Ahmed et al.^2^. A Maxwell nanofluid's erratic flow while being heated by Newtonian radiation was examined by Raza and Asad^3^. The effects of heat transfer on the free convection flow of a hybrid Maxwell nanofluid down an indefinite vertical channel were examined by Ahmed et al.^4^. By combining the effects of an electric and magnetic field with the effects of thermal and changeable heat radiation, Khan et al.^5^ research looked at the Maxwell nanofluid flow across a starching surface. Maxwell nanofluid flow across a stretching porous medium with the influence of magnetohydrodynamics was discussed mathematically by Mukhtar et al.^6^. The mixed convection flow of the Maxwell nanofluid with the impact and ion slip of the hall was examined by Ibrahim and Abneesa^7^. The Maxwell nanofluid flow across an infinite vertical with the influence of ramping and isothermal wall conditions was studied by Khan et al.^8^. The MHD Maxwell nanofluid flow across the porous stretched sheet with gyrotactic microorganisms is researched by Safdar et al.^9^ and is discussed theoretically and numerically. The temperature and mass characteristics of the Soret-Dufour model of magnetized Maxwell nanofluid flow across a shrinking inclined plane were examined by Parvin et al.^10^. Ahmad et al.^11^ examined the bio-convective Maxwell nanofluid flow via an exponentially stretched sheet with the convective boundary condition. Rasool et al.^12^ examined the Darcy-Forchheimer medium and heat radiation in the magnetohydrodynamic (MHD) Maxwell nanofluid flow confronted to a stretched surface. Alsallami et al.^13^ conducted a numerical analysis of the nanofluid flow across a heated rotating disc under the effects of Brownian motion, thermophoresis, and nonlinear radiation.

In a letter to Leibniz, L'Hospital posed an issue that led to the development of fractional calculus^14^. L'Hospital questioned anything about $D^{n} f(r)/Dr^{n}$ this letter. When L'Hospital inquired about the result of n = 1/2 Leibniz responded that it would initially seem to be a contradiction from which important insights would one day be drawn. Famous mathematicians including Euler, Laplace, Fourier, Lacroix, Abel, Riemann, and Liouville developed an interest in the subject after this conversation. They contributed to its growth. For a few decades, mathematicians were the only ones who had any knowledge of this topic. But in recent years, the idea of this topic has been expanded to various other disciplines in a number of different ways, including modeling speech signals^15–20^, modeling cardiac tissue electrode interface^21^, modeling sound wave propagation in solid permeable medium^22^, lateral and longitudinal governing of Sovran vehicle^23^, and so on. The most popular fractional derivatives were the Riemann–Liouville derivatives. These derivatives, however, had severe limitations and were only applicable to a specific class of issues. For instance, in the Riemann–Liouville fractional derivative, the constant does not result in zero when we take its derivative.

To address these shortcomings, Caputo develops a novel derivative, however the Caputo derivative's kernel remained singular. To address these concerns,^24^ developed a new exponential function-based fractional operator without a solitary kernel in 2015. The Laplace transformation also works with the Caputo-Fabrizio (CF) derivative.^25^ investigated the flow of a viscous fluid via an infinitely mobile plate. Due to its abstract nature, fractional calculus did not initially attract the attention of researchers. Its method has evolved over time from conceptual to practical, and as a result, it has gained popularity among scholars. Contrary to classical calculus, non-integer derivatives are much more prevalent in practically all scientific disciplines^26–29^.

The practical applications of local derivatives extend beyond engineering to include integrated circuits, electrochemistry, probability, curve fitting, and nuclear fusion^30–32^. Imran et al.^33^ observed the rheology of fractional Maxwell fluid in the presence of Newtonian heating effects while keeping in mind the stated relevance. The authors changed a non-local model into a local mathematical fractional order model using the CF operator. In a Maxwell fluid with a CF derivative, Khan et al.^34^ examined the evaluation of heat transfer over a fluctuating vertical plate. By generalizing it using the CF fractional derivative, Saqib et al.^35^ explored the free convection flow of a hybrid nanofluid with heat transfer.

Nanofluids have a significant impact on a variety of industrial sectors where heat transmission is essential due to their enhanced thermal conductivity properties. In order to improve the thermal conductivity of ordinary fluids, Choi^36^ created the contemporary theory of interruption of nanosized particles. Thermal insulation, energy production, nuclear reactor cooling, electricity processing, and cancer therapy are just a few of the many uses for nanofluids. Applications for nanofluids go beyond simply increasing the thermal conductivity of fluids; they also play a beneficial function in intelligent technology, drug delivery, disease diagnostics, food processing, and other areas^37^. The mechanical characteristics of nanoparticles were investigated by Guo et al.^38^ for brand-new applications in a variety of industries, such as surface engineering, tribology, and coating.

Burg et al.^39^ investigation of the fluid weights of individual cells, biomolecules, and nanoparticles was conducted. In a small shell-and-tube heat exchanger with and without a fin, Bahiraei and Monavari^40^ investigated the impact of different nanoparticle morphologies on the thermal–hydraulic performance of a boehmite nanofluid. Al_2_O_3_-water nanofluid was used in Mazaheri et al.^41^ investigation of the properties of a counter-flow four-layer microchannel heat exchanger. In their investigation of the pipe side, Bahiraei and Monavari^42^ used water as the cold fluid and a nanofluid with five distinct particle morphologies as the hot fluid. In a triple-tube heat exchanger, a new crimped-spiral rib with irreversibility properties was used by Bahiraei et al.^43^ in their study on thermal applications.

Oils are not always the greatest choice when it comes to lubricating parts. The lubricant may need to stick to a part in some situations. Regular maintenance is necessary to stop stains and damage caused by oil leaks, which is costly in terms of both time and money. Only the grease needs to be changed every 6 months, allowing one bearing to function until a roll change. More money has been saved by doing away with maintenance tasks like changing seal hearing. On the other hand, components that are difficult to obtain require lubrication. Grease-like semisolid lubricants, which share many characteristics with their fluid counterparts, are designed to stick to or adhere to the parts they are meant to lubricate. Grease is frequently used to lessen friction in machinery. Lubricating grease is made up of three ingredients: oil, thickener, and additives. The primary ingredients in grease formulations, base oil and additive kit, have a considerable impact on grease behavior. The thickening, which is also known as a sponge, keeps the lubricant in place. Grease can extend the life of equipment that is difficult to reach for multiple lubrications and worn sections that were previously lubricated with oil by keeping thicker films in wear-widened clearances. High-quality greases can lubricate uncommonly hard-to-reach components for extended periods of time without needing to be replenished on a regular basis. The centralized lubrication systems, electrical motors, bearings, truck wheel hubs for logging and mining equipment, construction, landscaping, and gearboxes are just a few of the sealed life applications that use these greases. In contemporary technologies including the oil business, sealing agents, the automotive industry, and the metalworking sector, grease has practical applications^44–47^. The tribological characteristics of conductive lubricating greases were explored by Fan et al.^48^ who also covered experimental methods including scanning electron microscopy to study friction processes. Grease qualities, especially those based on metal soap, depend not only on their composition but also on how the thickeners are prepared and mixed^49^.

From the above literature survey, it is evident that, no exact solutions are reported for Maxwell nanofluid flow using the CF fractional approach. To fill this gap, we have considered an open channel flow of Maxwell nanofluid together with heat and mass transfer. For this purpose, relevant constitutive equations have been used to model the problem in terms of classical PDEs and generalized using the Caputo-Fabrizio fractional derivative approach. The obtained fractional model is solved by jointly using two different mathematical tools, namely, the finite Fourier sine transform and the Laplace transform technique. More importantly, the current research focuses on using MoS_2_ nanoparticles in grease to improve mechanical properties such as weak machine friction reduction, low heat transfer power, low lubrication, and various other mechanical problems.

## Mathematical formulation

**Figure 1:**
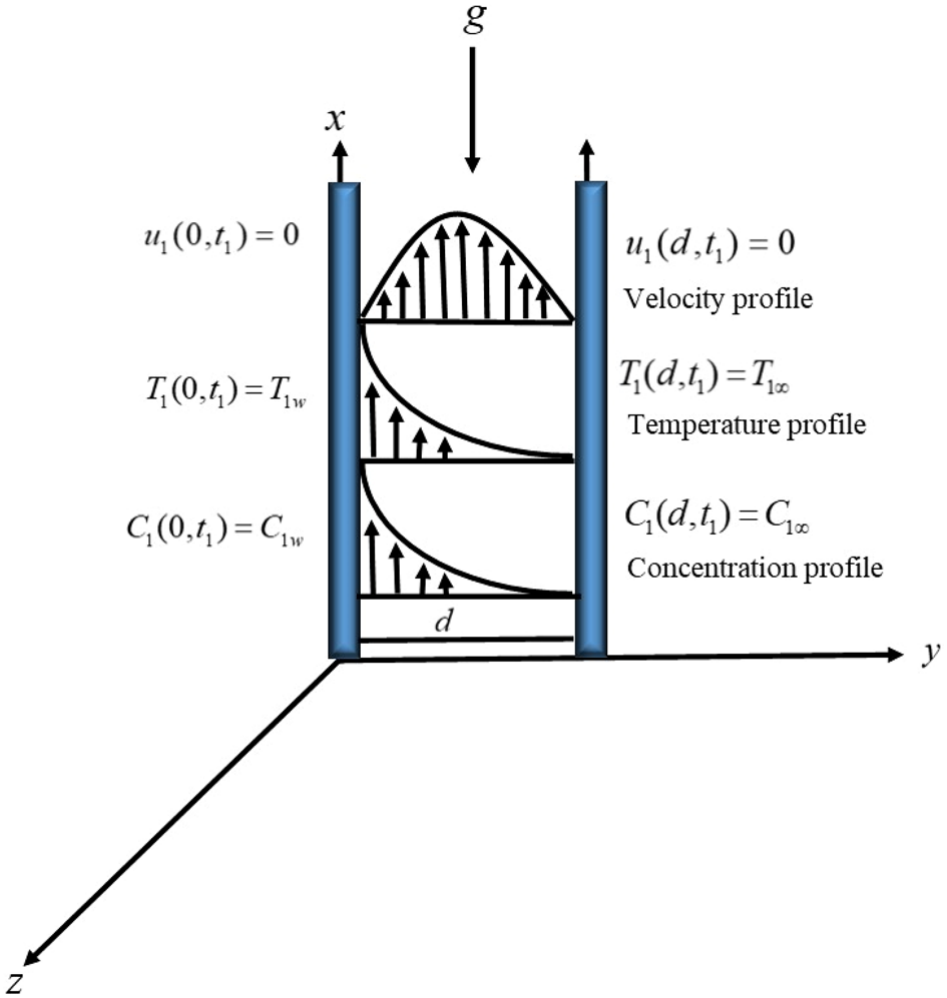
The physical configuration of the problem.

In the present work, we assumed viscoelastic Maxwell nanofluid flow between two vertical parallel plates separated by a distance of *d*. The fluid motion is considered in $x$ the direction in the presence of buoyancy force. To improve the rate of heat transfer, *MoS*_*2*_ nanoparticles are suspended uniformly within the grease, which is taken as a base fluid. Initially, both the plate and fluid are at rest with ambient temperature $T_{1\infty }$ and constant concentration $C_{1\infty }$. For t = 0^+^, the temperature and concentration of the left plate increased to T_1w_ and C_1w_. The governing equations of the given flow regime are as follows (Fig. 1):

In light of assumptions, the velocity, temperature and concentration fields are given as;

1 $$\overrightarrow {V} = \left( {u\left( {y,t} \right)\hat{i},0\hat{j},0\hat{k}} \right),$$

2 $$T = \left( {T\left( {y,t} \right),0,0} \right),$$

3 $$C = \left( {C\left( {y,t} \right),0,0} \right),$$

The continuity, momentum, heat and concentration equations in constitutive form are given as follow^50,51^:

4 $$\nabla \cdot \overrightarrow {V} = 0.$$

5 $$\rho \left[ {\frac{{\partial \!\mathop{V}\limits^{\rightharpoonup} }}{\partial t}} \right] = {\text{div}}{\mathbf{T}} + \rho\!\mathop{b}\limits^{\rightharpoonup}$$

6 $$\rho c_{p} \frac{\partial T}{{\partial t}} = k\nabla \times \nabla \times T,$$

7 $$\frac{\partial C}{{\partial t}} = D\nabla \times \nabla \times C.$$

Keeping in view the assumption and Eqs. (1)–(3), Eqs. (5)–(7) will take the form:

8 $$\begin{aligned} \rho_{nf} \left( {1 + \lambda_{1} \frac{\partial }{{\partial t_{1} }}} \right)\frac{{\partial u_{1} (y_{1} ,t_{1} )}}{{\partial t_{1} }} & = \mu_{nf} \frac{{\partial^{2} u_{1} (y_{1} ,t_{1} )}}{{\partial y_{1}^{2} }} + \left( {1 + \lambda_{1} \frac{\partial }{{\partial t_{1} }}} \right)\left( {\rho \beta_{T} } \right)_{nf} g\left( {T_{1} (y_{1} ,t_{1} ) - T_{1\infty } } \right) \\ & \;\; + \left( {1 + \lambda_{1} \frac{\partial }{{\partial t_{1} }}} \right)\left( {\rho \beta_{C} } \right)_{nf} g\left( {C_{1} - C_{1\infty } } \right), \\ \end{aligned}$$

9 $$\left( {\rho C_{p} } \right)_{nf} \frac{{\partial T_{1} }}{{\partial t_{1} }} = k_{nf} \frac{{\partial^{2} T_{1} }}{{\partial y_{1}^{2} }},$$

10 $$\frac{{\partial C_{1} }}{{\partial t_{1} }} = D_{nf} \frac{{\partial^{2} C_{1} }}{{\partial y_{1}^{2} }}.$$

Subject to the below imposed physical conditions:

11 $$\left. \begin{gathered} u_{1} (y_{1} ,0) = 0,\,\,T_{1} (y_{1} ,0) = T_{1\infty } ,\,\,C_{1} (y_{1} ,0) = C_{1\infty ,} \hfill \\ u_{1} (0,t) = 0,\,\,\,T_{1} (0,t_{1} ) = T_{1w} ,\,\,C(0,t_{1} ) = C_{1w,} \hfill \\ u_{1} (\infty ,t_{1} ) = 0,\,\,\,\,T_{1} (d,t_{1} ) = T_{1\infty } ,\,\,\,\,C_{1} (d,t_{1} ) = C_{1\infty ,} \hfill \\ \left. {\frac{{\partial u_{1} (y_{1} ,t_{1} )}}{{\partial t_{1} }}} \right|_{{t_{1} = 0}} = 0 \hfill \\ \end{gathered} \right\}.$$

In the above system of equations, the velocity component of the Maxwell nanofluid along the *x-*axis is denoted by $u_{1}$, $T_{1}$ is the fluid temperature, $T_{1\infty }$ shows the ambient temperature, and $T_{1w}$ is the wall temperature. The thermophysical properties of the grease and *MoS*_*2*_ nanoparticles are given in Table 1.

**Table 1:** Thermophysical properties of grease and *MoS*_*2*_^44–49^:

Material	$\rho \;({\text{km}}^{ - 3} )$	$c_{p} \;({\text{JKg}}^{ - 1} {\text{K}}^{ - 1} )$	$k\;({\text{wm}}^{ - 1} {\text{K}}^{ - 1} )$	$\beta \; \times \;10^{ - 5} \;({\text{K}}^{ - 1} )$
Grease	883	2100	0.1450	0.00001616
$MoS_{2}$	506×10^3^	397.21	904.4	2.8424

For nanofluids, expression of $\rho_{nf} ,\,\left( {\rho \beta } \right)_{nf} ,\left( {\rho c_{p} } \right)_{nf} ,\,\,k_{nf} ,\,$ is given by^20^.

12 $$\left. \begin{gathered} \rho_{nf} = \rho_{f} (1 - \phi ) + \phi \rho_{s} ,\,\left( {\rho \beta_{T} } \right)_{nf} = (\rho \beta_{T} )_{f} (1 - \phi ) + \phi (\rho \beta_{T} )_{s} ,\, \hfill \\ \left( {\rho \beta_{C} } \right)_{nf} = \phi (\rho \beta_{C} )_{s} + (\rho \beta_{C} )_{f} (1 - \phi ),\,(\rho c_{p} )_{nf} = (1 - \phi )(\rho c_{p} )_{f} + \phi (\rho c_{p} )_{s} ,\, \hfill \\ D_{nf} {\text{ = D}}_{f} \left( {1 - \phi } \right){\text{, k}}_{nf} = k_{f} \left[ {\frac{{k_{s} + 2k_{f} - 2\phi (k_{f} - k_{s} )}}{{k_{s} + 2k_{f} + \phi (k_{f} - k_{s} )}}} \right],\,\mu_{nf} = \mu_{f} (1 - \phi )^{ - 2.5} . \hfill \\ \end{gathered} \right\}.$$

Nondimensional quantities are:

13 $$\left. {w = \frac{{u_{1} }}{{U_{0} }}, \, \zeta = \frac{{y_{1} }}{d},\,\,\tau = \frac{{t_{1} U_{0} }}{d},\, \, \theta = \frac{{T_{1} - T_{1\infty } }}{{T_{1w} - T_{1\infty } }},\,\Phi = \frac{{C_{1} - C_{1\infty } }}{{C_{1w} - C_{1\infty } }}.} \right\}.$$

Using Eq. (13), the dimensionless forms of Eqs. (8)–(11) are given as follows:

14 $$A_{2} \left( {1 + \lambda \frac{\partial }{\partial \tau }} \right)\frac{\partial w(\zeta ,\tau )}{{\partial \tau }} = \frac{{\partial^{2} w(\zeta ,\tau )}}{{\partial \zeta^{2} }} + A\left( {1 + \lambda \frac{\partial }{\partial \tau }} \right)Gr\Theta + A_{1} \left( {1 + \lambda \frac{\partial }{\partial \tau }} \right)Gm\Phi ,$$

15 $$\frac{\partial \theta (\zeta ,\tau )}{{\partial \tau }} = \frac{1}{\psi }\frac{{\partial^{2} \Theta (\zeta ,\tau )}}{{\partial \zeta^{2} }},$$

16 $$\frac{\partial \Phi (\zeta ,\tau )}{{\partial \tau }} = \frac{1}{\varphi }\frac{{\partial^{2} \Phi (\zeta ,\tau )}}{{\partial \zeta^{2} }}.$$

Physical conditions in dimensionless form:

17 $$\left. \begin{gathered} w(w,0) = 0,\,\,\,\theta (w,0) = 0,\,\,\,\Phi (w,0) = 0, \hfill \\ w(0,\tau ) = 0,\,\,\,\,\theta (0,\tau ) = 1,\,\,\,\,\,\Phi (0,\tau ) = 1, \hfill \\ w(1,\tau ) = 0,\,\,\,\theta (1,\tau ) = 0,\,\,\,\,\,\Phi (1,\tau ) = 0, \hfill \\ \left. {\frac{\partial w(y,\tau )}{{\partial t}}} \right|_{\tau = 0} = 0. \hfill \\ \end{gathered} \right\}.$$

Were

$$\lambda = \frac{{\lambda_{1} U_{0} }}{d}$$, $$\ell = (1 - \phi ) + \phi \frac{{\rho_{s} }}{{\rho_{f} }}$$, $$\ell_{1} = \left( {1 - \phi } \right) + \phi \frac{{\left( {\rho \beta_{T} } \right)_{s} }}{{\left( {\rho \beta_{T} } \right)_{f} }},$$ $$\ell_{2} = \left( {1 - \phi } \right) + \phi \frac{{\left( {\rho \beta_{c} } \right)_{s} }}{{\left( {\rho \beta_{c} } \right)_{f} }},$$ $$\ell_{3} = \frac{1}{{\left( {1 - \phi } \right)^{2.5} }}$$, $$Gr = \frac{{g\beta_{T} \rho d^{2} \left( {T_{w} - T_{d} } \right)}}{{U_{0} \mu }}$$, $$Gm = \frac{{g\beta_{C} \rho d^{2} \left( {C_{w} - C_{d} } \right)}}{{U_{0} \mu }}$$, $${\text{Re}} = \frac{{U_{0} d}}{\upsilon }$$, $$A = Gr\ell_{1} \ell_{3}$$, $$A_{1} = Gm\ell_{2} \ell_{3}$$, $$A_{2} = \ell \ell_{3} {\text{Re}}$$, $$\Pr = \frac{{\mu C_{p} }}{k}$$, $$\psi = \frac{{\Pr {\text{Re}} \phi_{2} }}{{\phi_{1} }}$$, $${\text{Sc = }}\frac{{\nu_{f} }}{{D_{f} }}$$, $$\varphi = \frac{{Sc{\text{Re}} }}{1 - \phi }$$.

## Exact solutions of the problem

By applying the Caputo-Fabrizio (CF) time fractional derivative, Eqs. (14)–(16) will take the following shape:

18 $$\begin{aligned} A_{2} \left( {1 + \lambda {}^{CF}\wp_{\tau }^{\alpha } } \right){}^{CF}\wp_{\tau }^{\alpha } w(\zeta ,\tau ) & = \frac{{\partial^{2} w(\zeta ,\tau )}}{{\partial \zeta^{2} }} + A\left( {1 + \lambda {}^{CF}\wp_{\tau }^{\alpha } } \right)Gr\theta (\zeta ,\tau ) \\ & \;\;\; + A_{1} \left( {1 + \lambda {}^{CF}\wp_{\tau }^{\alpha } } \right)Gm\Phi (\zeta ,\tau ), \\ \end{aligned}$$

19 $${}^{CF}\wp_{t}^{\alpha } \theta (\zeta ,\tau ) = \frac{1}{\psi }\frac{{\partial^{2} \theta (\zeta ,\tau )}}{{\partial \zeta^{2} }}.$$

20 $${}^{CF}\wp_{t}^{\alpha } \Phi (\zeta ,\tau ) = \frac{1}{\varphi }\frac{{\partial^{2} \Phi (\zeta ,\tau )}}{{\partial \xi^{2} }},$$

where ${}^{CF}D_{t}^{\alpha } \left( . \right)$ is the CF time fractional operator, which is given by^52^:

21 $${}^{CF}D_{t}^{\alpha } f\left( t \right) = \frac{M(\alpha )}{{(1 - \alpha )}}\int\limits_{0}^{t} {\exp \left[ { - \frac{\alpha (t - \tau )}{{1 - \alpha }}} \right]f^{\prime}(\tau )d\tau } .$$

where $M(\alpha )$ is a normalization function such that $M(0) = M(1) = 1$.

### Solution of the energy equation

By applying the Laplace transform technique to the Eq. (19) and incorporate IC’s and BC’s, we get:

22 $$\frac{{d^{2} \overline{\theta }}}{{d\xi^{2} }} = \frac{{a\psi s\overline{\theta }\left( {\zeta ,s} \right)}}{{s + a_{1} }},$$

By applying the finite Fourier sine transform to the Eq. (22), we get:

23 $$\overline{\Theta }_{F} \left( {n,s} \right) = \left( {\frac{n\pi }{{a_{0} \psi + n\pi }}} \right)\left( {\frac{{a_{1} }}{{a_{2} s}} - \frac{{a_{1} - a_{2} }}{{a_{2} \left( {s + a_{2} } \right)}}} \right),$$

By applying inverse Laplace transform on Eq. (23), we arrived at:

24 $$\Theta_{F} \left( {n,\tau } \right) = \frac{{1 - \left( { - 1} \right)^{n} }}{n\pi } - \frac{{\left( { - 1} \right)^{n + 1} }}{n\pi } - a_{3} \exp \left( { - a_{2} \tau } \right),$$

where $$a_{2} = \frac{{a_{1} \left( {n\pi } \right)^{2} }}{a\psi + n\pi }$$, $$a_{3} = \left( {\frac{n\pi }{{a\psi + n\pi }}} \right)\left( {\frac{{a_{1} - a_{2} }}{{a_{2} }}} \right)$$, $${\text{a}} = \frac{1}{1 - \alpha },\,\,{\text{a}}_{1} = \frac{\alpha }{1 - \alpha }$$

By applying inverse finite Fourier sine transform to Eq. (24), we get:

25 $$\theta \left( {\zeta ,\tau } \right) = 1 - \zeta - 2\sum\limits_{n = 1}^{\infty } {a_{3} \exp \left( { - a\tau } \right)\sin \left( {n\pi \zeta } \right)} .$$

### Solution of the concentration equation

By applying the Laplace transform technique to Eq. (20) and incorporating ICs and BCs, we obtain:

26 $$\frac{{d^{2} \overline{\Phi }\left( {\zeta ,p} \right)}}{{d\zeta^{2} }} = \frac{{a\varphi s\overline{\Phi }\left( {\zeta ,p} \right)}}{{p + a_{1} }},$$

By applying the finite Fourier sine transform to Eq. (26), we obtain:

27 $$\overline{\Phi }_{F} \left( {n,p} \right) = \left( {\frac{n\pi }{{a\varphi - n\pi }}} \right)\left( {\frac{{a_{1} }}{{a_{2} p}} - \frac{{a_{1} - a_{2} }}{{a_{2} \left( {p + a_{2} } \right)}}} \right),$$

By applying the inverse Laplace transform to Eq. (27), we obtain:

28 $$\Phi_{F} \left( {n,\tau } \right) = \frac{{1 - \left( { - 1} \right)^{n} }}{n\pi } - \frac{{\left( { - 1} \right)^{n + 1} }}{n\pi } - a_{4} \exp \left( { - a\tau } \right),$$

Here,

$$a_{4} = \frac{{\left( {a_{1} - a_{2} } \right)n\pi }}{{a_{2} \left( {a\varphi - n\pi } \right)}}$$

By applying the inverse finite Fourier sine transform to Eq. (28), we obtain:

29 $$\Phi \left( {\zeta ,\tau } \right) = 1 - \zeta - 2\sum\limits_{n = 1}^{\infty } {a_{3} \exp \left( { - a_{2} t} \right)\sin \left( {n\pi \zeta } \right)} .$$

### Solution of the momentum equation

Applying the Laplace transform to Eq. (14) and incorporating ICs and BCs, we obtain:

30 $$\begin{aligned} \frac{{ap\overline{w}(\zeta ,p)\left( {1 + \lambda p} \right)A_{2} }}{p + a} & = \frac{{d^{2} \overline{w}}}{d\zeta } + \left( {1 + \frac{ap}{{p + a}}} \right)\left( {\frac{n\pi }{{am + n\pi }}} \right)\left( {\frac{{p + a_{1} }}{{p\left( {p + a_{2} } \right)}}} \right) \\ \, & \,\,\, + \left( {1 + \frac{ap}{{p + a}}} \right)\left( {\frac{n\pi }{{am_{2} + n\pi }}} \right)\left( {\frac{{p + a_{1} }}{{p\left( {p + a_{2} } \right)}}} \right), \\ \end{aligned}$$

By applying a sine finite Fourier transform to Eq. (30), we obtain

31 $$\begin{aligned} \overline{w}_{F} \left( {n,p} \right) & = \frac{1}{p}\left( {\frac{{a_{1} A_{13} A_{18} }}{{a_{2} A_{10} A_{11} }}} \right) + \frac{1}{{p + a_{2} }}\left( {\frac{{\left( {a_{1} A_{13} - a_{1} a_{2} - a_{2} A_{13} - a_{2}^{2} } \right)A_{18} }}{{a_{2} \left( {A_{10} - a_{2} } \right)\left( { - A_{11} + a_{2} } \right)}}} \right) \\ & \;\;\; + \,\frac{1}{{p + A_{10} }}\left( {\frac{{\left( {a_{1} A_{10} + A_{10}^{2} - a_{1} A_{13} + A_{10} A_{13} } \right)A_{18} }}{{A_{10} \left( {A_{10} - A_{11} } \right)\left( {A_{10} - a_{2} } \right)}}} \right) \\ & \;\;\; + \,\frac{1}{{p + A_{11} }}\left( {\frac{{\left( { - a_{1} A_{11} + A_{11}^{2} + a_{1} A_{13} - A_{11} A_{13} } \right)A_{18} }}{{A_{11} \left( {A_{10} - A_{11} } \right)\left( {A_{11} - a_{2} } \right)}}} \right), \\ \end{aligned}$$

By applying the inverse Laplace transform to Eq. (31), we obtain the following form:

32 $$\begin{aligned} w_{F} \left( {n,\tau } \right) & = \frac{{a_{1} A_{13} A_{18} }}{{a_{2} A_{10} A_{11} }} + \left( {\frac{{\left( {a_{1} A_{13} - a_{1} a_{2} - a_{2} A_{13} - a_{2}^{2} } \right)A_{18} }}{{a_{2} \left( {A_{10} - a_{2} } \right)\left( { - A_{11} + a_{2} } \right)}}} \right)e^{{ - a_{2} \tau }} \\ & \,\,\, + \left( {\frac{{\left( {a_{1} A_{10} + A_{10}^{2} - a_{1} A_{13} + A_{10} A_{13} } \right)A_{18} }}{{A_{10} \left( {A_{10} - A_{11} } \right)\left( {A_{10} - a_{2} } \right)}}} \right)e^{{ - A_{10} \tau }} \\ & \,\,\; + \left( {\frac{{\left( { - a_{1} A_{11} + A_{11}^{2} + a_{1} A_{13} - A_{11} A_{13} } \right)A_{18} }}{{A_{11} \left( {A_{10} - A_{11} } \right)\left( {A_{11} - a_{2} } \right)}}} \right)e^{{ - A_{10} \tau }} , \\ \end{aligned}$$

Now, apply the inverse finite Fourier sine transform to Eq. (32) to obtain the following form:

33 $$w\left( {\zeta ,\tau } \right) = 2\sum\limits_{n = 1}^{\infty } {\left( \begin{gathered} \frac{{a_{1} A_{13} A_{18} }}{{a_{2} A_{10} A_{11} }} + \left( {\frac{{\left( \begin{gathered} a_{1} A_{13} - a_{1} a_{2} \hfill \\ - a_{2} A_{13} - a_{2}^{2} \hfill \\ \end{gathered} \right)A_{18} }}{{a_{2} \left( {A_{10} - a_{2} } \right)\left( { - A_{11} + a_{2} } \right)}}} \right)e^{{ - a_{2} \tau }} \hfill \\ + \left( {\frac{{\left( {a_{1} A_{10} + A_{10}^{2} - a_{1} A_{13} + A_{10} A_{13} } \right)A_{18} }}{{A_{10} \left( {A_{10} - A_{11} } \right)\left( {A_{10} - a_{2} } \right)}}} \right)e^{{ - A_{10} \tau }} \hfill \\ + \left( {\frac{{\left( { - a_{1} A_{11} + A_{11}^{2} + a_{1} A_{13} - A_{11} A_{13} } \right)A_{18} }}{{A_{11} \left( {A_{10} - A_{11} } \right)\left( {A_{11} - a_{2} } \right)}}} \right)e^{{ - A_{10} \tau }} \hfill \\ \end{gathered} \right)} \sin \left( {n\pi \zeta } \right).$$

Introduced some constant

$$\begin{aligned} A_{5} & = aA_{2} ,\,A_{6} = \lambda A_{5} ,\,A_{7} = A_{5} + \left( {n\pi } \right)^{2} ,\,A_{8} = a_{1} \left( {n\pi } \right)^{2} ,\,A_{9} = \sqrt {\frac{{A_{7} - 4A_{8} }}{2}} , \\ A_{10} & = \frac{{A_{7} }}{2} + A_{9} ,\,A_{11} = \frac{{A_{7} }}{2} - A_{9} ,\,A_{12} = a + 1,\,A_{13} = \frac{{a_{1} }}{{A_{12} }},\,A_{14} = A_{12} A,\,A_{15} = A_{12} A_{1} , \\ A_{16} & = \frac{{A_{14} \left( {n\pi } \right)}}{am + n\pi },\,A_{17} = \frac{{A_{15} \left( {n\pi } \right)}}{{am_{2} + n\pi }},\,A_{18} = A_{16} + A_{17} . \\ \end{aligned}$$

### Nusselt number

The dimensional form of the Nusselt number for a Maxwell nanofluid is given by^8^:

34 $$Nu = - \frac{{k_{nf} }}{{k_{f} }}\left( {\frac{d}{{T_{1w} - T_{1\infty } }}} \right)\left. {\frac{{\partial T_{1} }}{{\partial y_{1} }}} \right|_{{y_{1} = 0}}$$

By using Eq. (13), the dimensionless form of Eq. (34) becomes:

35 $$Nu = - \left. {\frac{{k_{nf} }}{{k_{f} }}\frac{\partial \theta }{{\partial \zeta }}} \right|_{\zeta = 0}$$

### Sherwood number

The dimensional form of the Sherwood number for a Maxwell nanofluid is given by^8^:

36 $$S_{h} = - D_{nf} \left( {\frac{d}{{C_{1w} - C_{1\infty } }}} \right)\left. {\frac{{\partial C_{1} }}{{\partial y_{1} }}} \right|_{{y_{1} = 0}}$$

By using Eq. (13), the dimensionless form of Eq. (36) becomes:

37 $$S_{h} = - \left. {D_{nf} \frac{\partial \Phi }{{\partial \zeta }}} \right|_{\zeta = 0}$$

### Skin friction

The dimensional form of nonzero shear stress for Maxwell fluid is given as:

38 $$\tau_{xy}^{*} = \frac{{\mu_{f} }}{{\left( {1 + \lambda \frac{\partial }{\partial \tau }} \right)}}\frac{{\partial u_{1} }}{{\partial y_{1} }},$$

For a Maxwell nanofluid, Eq. (38) takes the following form:

39 $$\tau_{xy}^{*} = \frac{{\mu_{nf} }}{{\left( {1 + \lambda \frac{\partial }{\partial \tau }} \right)}}\frac{{\partial u_{1} }}{{\partial y_{1} }},$$

By using Eq. (12) and Eq. (13), the dimensionless form of Eq. (39) becomes:

40 $$Sf_{lp} = \frac{1}{{\left( {1 - \phi } \right)^{2.5} }}\frac{1}{{\left( {1 + \lambda \frac{\partial }{\partial \tau }} \right)}}\frac{\partial w}{{\partial \zeta }},$$

where $\tau_{xy} = \frac{{\tau_{xy}^{*} \,d}}{{\mu_{f} U_{0} }}$ is the dimensionless form of nonzero shear stress and $\lambda = \frac{{\lambda_{1} U_{0} }}{d}$ is the dimensionless Maxwell parameter.

This problem is considered for the flow of Maxwell nanofluids through vertical plates. Therefore, the skin friction on the left and right plates is given by:

41 $$\begin{gathered} Sf_{lp} = \frac{1}{{\left( {1 - \phi } \right)^{2.5} }}\frac{1}{{\left( {1 + \lambda \frac{\partial }{\partial \tau }} \right)}}\left. {\frac{\partial w}{{\partial \zeta }}} \right|_{\zeta = 0} \hfill \\ \hfill \\ \end{gathered}$$

42 $$Sf_{rp} = \frac{1}{{\left( {1 - \phi } \right)^{2.5} }}\frac{1}{{\left( {1 + \lambda \frac{\partial }{\partial \tau }} \right)}}\left. {\frac{\partial w}{{\partial \zeta }}} \right|_{\zeta = 1}$$

where $Sf_{lp}$ and $Sf_{rp}$ denote the skin friction at the left and right plates, respectively.

## Special case

The obtained results given in Eqs. (25), (29) and (33) can be reduced to the results published by Khalid et al*.*^53^ by ignoring $\lambda \to 0$, $\alpha \to 1$ and mass Grashof number *Gm.*

## Results and discussion

**Figure 2:**
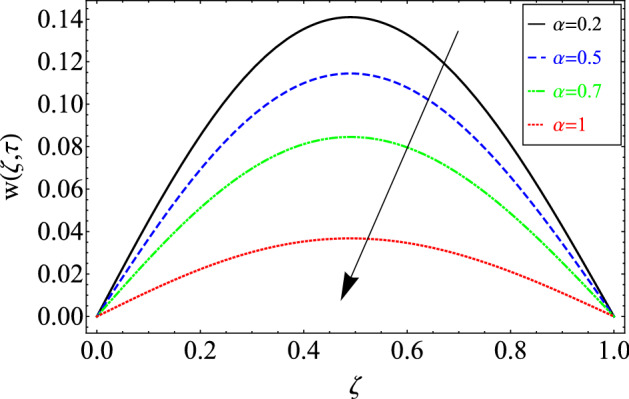
The effect of different values of $\alpha$ on velocity distribution of Maxwell nanofluid, when ${\text{Re}} = 10$, $\tau = 0.5$, $\phi = 0.02$, $Gr = 0.05,$ $Gm = 0.5,$ $\Pr = 6300,$ $Sc = 15$ and $\lambda = 0.5$.

**Figure 3:**
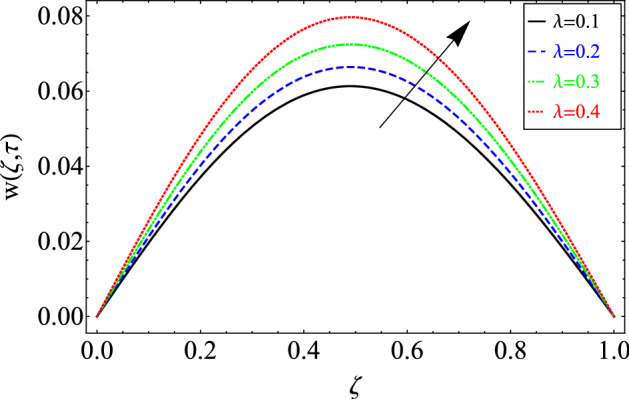
The effect of different values of $\lambda$ on velocity distribution of Maxwell nanofluid, when ${\text{Re}} = 10$, $\alpha = 0.7$, $\tau = 0.5$, $\phi = 0.02$, $Gr = 0.05\,$, $\Pr = 6300$, $Sc = 15$ and $Gm = 0.5$.

**Figure 4:**
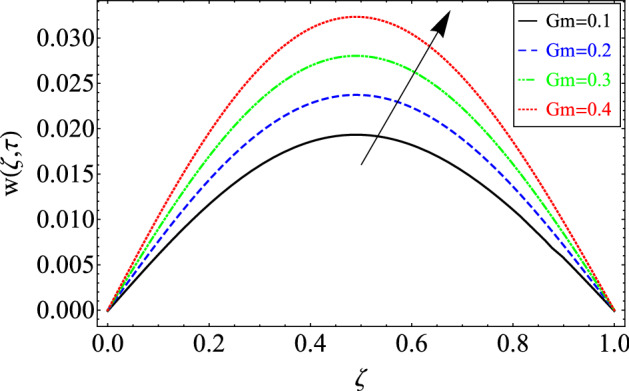
The effect of different values of *Gm* on velocity distribution of Maxwell nanofluid, when ${\text{Re}} = 10$, $\alpha = 0.7$, $\tau = 0.5$, $\phi = 0.02$, $Gr = 0.05\,$, $\Pr = 6300$, $Sc = 15$ and $\lambda = 0.5$.

**Figure 5:**
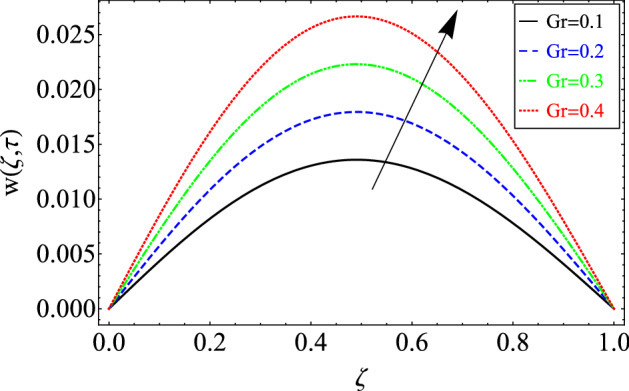
The effect of different values of *Gr* on velocity distribution of Maxwell nanofluid, when ${\text{Re}} = 10$, $\alpha = 0.7$, $\tau = 0.5$, $\phi = 0.02$, $Gm = 0.5$, $\Pr = 6300$, $Sc = 15$ and $\lambda = 0.5$.

**Figure 6:**
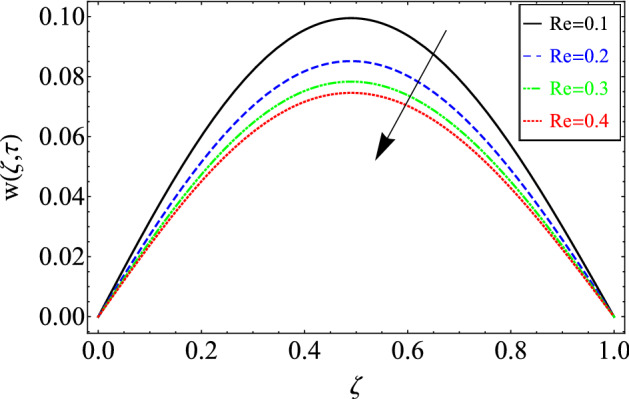
The effect of different values of *Re* on velocity distribution of Maxwell nanofluid, when $\alpha = 0.7$, $\tau = 0.5$, $\phi = 0.02$, $Gm = 0.5,$ $Gr = 0.05$, $\Pr = 6300$, $Sc = 15$ and $\lambda = 0.5$.

**Figure 7:**
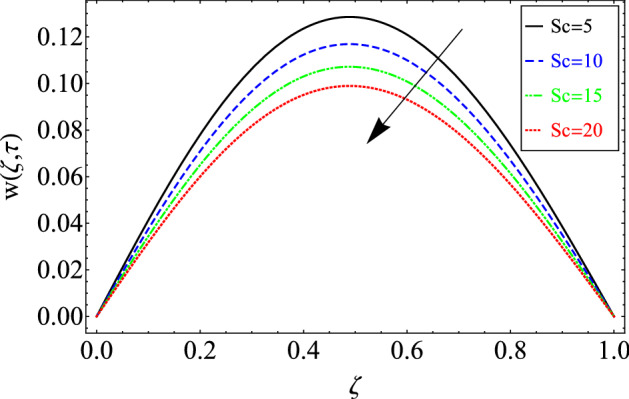
The effect of different values of *Sc* on velocity distribution of Maxwell nanofluid, when $$\alpha = 0.7$$, $$\tau = 0.5$$, $$\phi = 0.02$$,$$Gm = 0.5,$$$$Gr = 0.05$$, $$\Pr = 6300$$, $${\text{Re}} = 10$$ and $$\lambda = 0.5$$.

**Figure 8:**
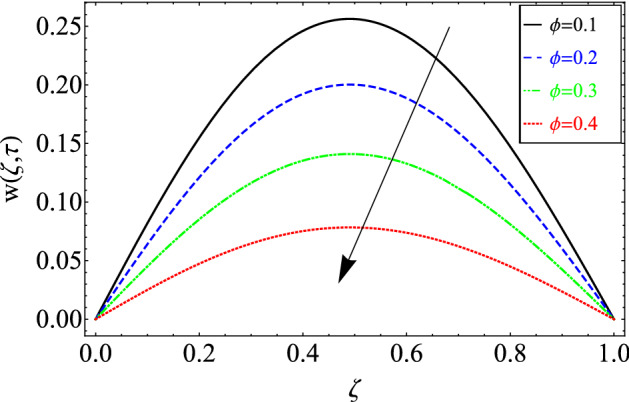
The effect of different value of $\phi$ on velocity distribution of Maxwell nanofluid, when $\alpha = 0.7$, $\tau = 0.5$, $Sc = 15$,$Gm = 0.5,$ $Gr = 0.05$, $\Pr = 6300$, ${\text{Re}} = 10$ and $\lambda = 0.5$.

**Figure 9:**
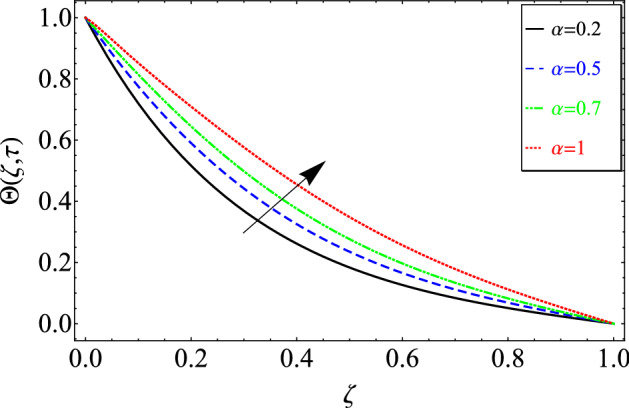
The effect of different values of $\alpha$ on temperature distribution of Maxwell nanofluid, when $\phi = 0.02$ and $\Pr = 6300$.

**Figure 10:**
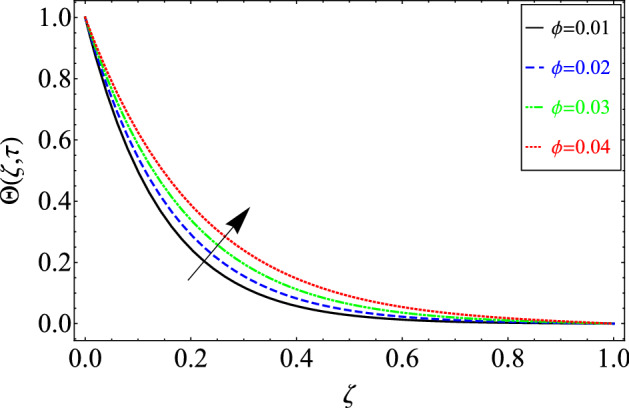
The effect of different values of $\phi$ on Temperature distribution of Maxwell nanofluid, when $\alpha = 0.7$ and $\Pr = 6300$.

**Figure 11:**
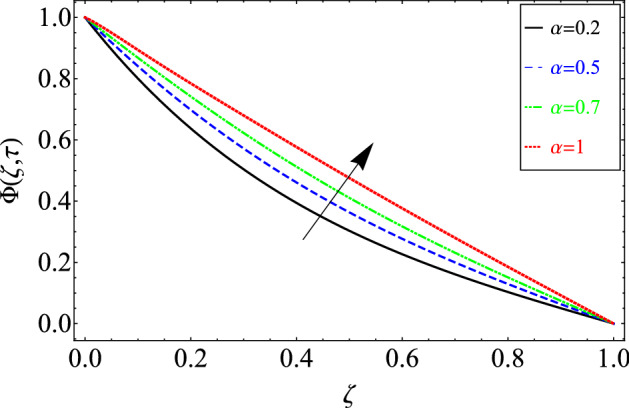
The effect of different values of $\alpha$ on concentration distribution of Maxwell nanofluid, when $\phi = 0.02$ and $Sc = 20$.

**Figure 12:**
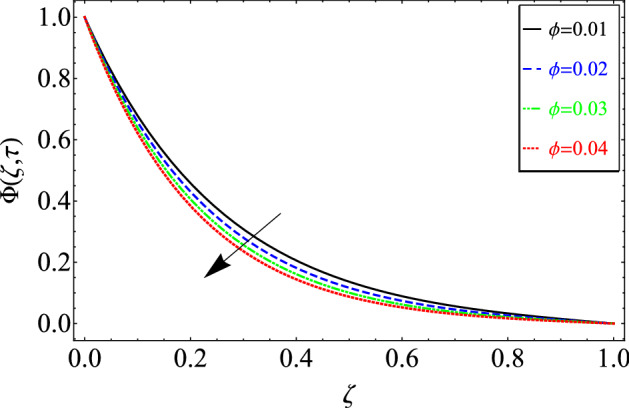
The effect of different values of $\phi$ on concentration distribution of Maxwell nanofluid, when $\alpha = 0.7$ and $Sc = 20$.

This section includes the definitive explanation of the Maxwell fluid model free convection flow. Approaching nondimensional variables is applied to make the PDE system dimensionless. The fractional Maxwell fluid model has been developed by implementing the CF time-fractional derivative. The joint use of the Laplace and Sine Finite Fourier techniques evaluated exact results for velocity, temperature, and concentration profiles. The thermophysical properties of *MoS*_*2*_ nanoparticles and grease are given in Table 1. The graphical analysis shows various embedded parameters $\alpha ,\,\tau ,\,\lambda ,\,Gm,\,Gr,\,{\text{Re}} ,\,Sc$ and $\phi$ on velocity, temperature, and concentration profiles. Furthermore, in Figs. 2, 3, 4, 5, 6, 7 and 8, the impact of sundry parameters on the velocity distribution is shown. The impact of sundry parameters on the temperature profile is shown graphically in Figs. 9 and 10. Finally, the impact of embedded parameters on the concentration distribution is shown graphically in Figs. 11 and 12.

Figure 2 is plotted to analyze flow rheology in response to fractional parameters $\alpha$. The prime benefit of the fractional model is that it provides more than one fluid layer for the investigation of fluid behavior. It gives the experimentalist and researchers more options for comparing their research to the fractional model, which is impossible by a classical mathematical model.

The velocity of the Maxwell nanofluid against the material parameter $\lambda$ is portrayed in Fig. 3. It can be seen from the expression of the material parameter that directly relates to the fluid's viscosity. Therefore, by raising the values of $\lambda$, the viscous forces increase, which leads to a decrease in fluid motion.

The impact of mass Grashof number $Gm$ on the velocity field has been plotted in Fig. 4. From the figure it can be seen clearly that velocity profile enhances for greater magnitude of *Gm.* This trend in the velocity field is physically true because when the value of *Gm* increases the concentration level near the plate increase and we know that fluid moves from higher concentration area to lower concentration area, therefore increasing trend has been observed.

The up shots of $Gr$ on the velocity field of grease have been plotted in Fig. 5. Increasing behavior has also been noticed for the increasing values of *Gr*. Physically, this trend is true because the greater magnitude of *Gr* weakens the boundary layer of the fluid and produces bouncy forces in the fluid. Due to these effects, the fluid motion accelerates.

The effect of Reynold number *Re* on velocity profile has been portrayed in Fig. 6. It can be seen from the figure that velocity profile shows decreasing trend for greater magnitude of *Re.* Physically, *Re* shows the relation between inertial forces and viscous forces. As the value of *Re* increase the viscous forces in the fluid enhance as a result the momentum boundary become thick and slow down the fluid.

Figure 7 shows Sc influence on the nanofluid velocity of Maxwell. The Maxwell nanofluid velocity is investigated by increasing Sc. Since Sc is the ratio of mass diffusion to viscous forces, raising Sc increases viscous forces and reduces mass diffusion, which reduces velocity.

Figure 8 depicts the variance in the velocity profile over a variety of different values of $\phi$. This figure shows that increasing the values of $\phi$ decreases its velocity. The reason for the decrease in velocity is that when $\phi$ rises, the fluid viscosity increases, resulting in the retardation of velocity.

Figure 9 displays the impact of fractional parameter $\alpha$ on temperature. This figure also shows the behavior of the temperature distribution for classical order by taking $\alpha = 1$ as well as fractional order $0 < \alpha < 1$ compared to classical models. The fractional model is more generalized, more effective for describing the memory effect, and provides a wide range of solutions. Compared to the classical Maxwell nanofluid model, the fractional-order Maxwell nanofluid model provides a better explanation of heat transfer by varying degrees.

Figure 10 shows the effect of $\phi$ on the temperature distribution. An increase in the temperature distribution is noticed for increasing values of *ϕ.* Regular grease has low thermal conductivity and lubrication properties. $MoS_{2}$ has a high thermal conductivity, which will increase the heat transfer rate of regular grease and the thermal conductivity of regular grease. As $MoS_{2}$ is also used as a dry lubricant, it will also increase the lubricity of regular grease.

Figure 11 shows the time fractional order parameter impacts on the concentration distribution. This figure shows the behavior of the concentration distribution for classical order $\alpha = 1$ and fractional order $0 < \alpha < 1.$ The same trend has been noticed in response to the fractional order parameter, as we discussed in Fig. 9.

Figure 12 shows the impact of $\phi$ on the concentration distribution. As seen in the figure, the concentration distribution decreases with increasing values of and. The reason for this phenomenon is that when the concentration distribution decreases, viscous forces rise.

**Figure 13:**
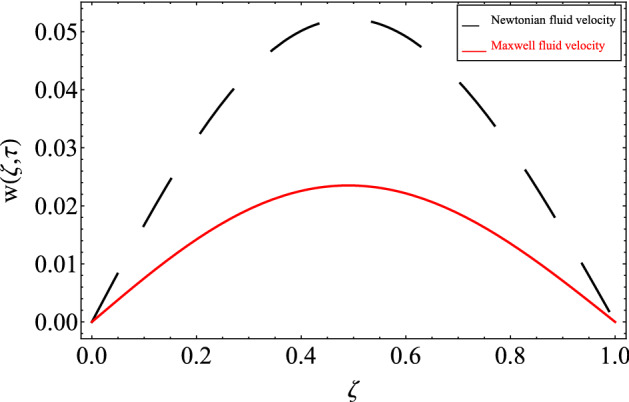
Comparison of the present results with the published results of Khalid et al.^53^.

Figure 13 compares our findings to the findings of the published article of Khalid, et al.^53^ to validate our obtained solutions. From this figure, our results matched the results of Khalid et al.^53^ by taking $\lambda \to 0$, $\alpha \to 1$ and $Gm \to 0$.

The variation in skin friction on the lower and upper plates is shown in Tables 2 and 3. These tables display the effects of skin friction for fractional and classical Maxwell nanofluid models along with other physical parameters. Tables 4 and 5 depict the Nusselt and Sherwood number variations, respectively, for distinct values of $\phi$. The heat transfer rate increases to 12.38%, and the mass distribution decreases to 2.14% by adding nanoparticles up to 4%.

**Table 2:** Skin friction of grease based *MoS*_*2*_ Maxwell nanofluid on the left plate.

$\lambda$	$Gm$	$Gr$	$\Pr$	Re	$\alpha$	$\tau$	$\phi$	$Sc$	$C_{f}^{\alpha }$	$C_{f}^{classical}$
*0.2*	*1.5*	*1.5*	6000	0.7	0.6	1.7	0.02	10		
*0.3*	*1.5*	*1.5*	6000	0.7	0.6	1.7	0.02	10	8.598	25.549
*0.2*	*2.5*	*1.5*	6000	0.7	0.6	1.7	0.02	10	9.581	15.622
*0.2*	*1.5*	*2.5*	6000	0.7	0.6	1.7	0.02	10	28.659	50.764
*0.2*	*1.5*	*1.5*	6500	0.7	0.6	1.7	0.02	10	6.461	20.192
*0.2*	*1.5*	*1.5*	6000	0.8	0.6	1.7	0.02	10	9.206	23.556
*0.2*	*1.5*	*1.5*	6000	0.7	0.7	1.7	0.02	10	14.039	21.746
*0.2*	*1.5*	*1.5*	6000	0.7	0.6	1.8	0.02	10	10.132	25.336
*0.2*	*1.5*	*1.5*	6000	0.7	0.6	1.7	0.04	10	13.713	34.406
*0.2*	*1.5*	*1.5*	6000	0.7	0.6	1.7	0.02	15	8.234	22.476

**Table 3:** Skin friction of grease based *MoS*_*2*_ Maxwell nanofluid on the right plate.

$\lambda$	$Gm$	$Gr$	$\Pr$	Re	$\alpha$	$\tau$	$\phi$	$Sc$	$C_{f}^{\alpha }$	$C_{f}^{classical}$
0.2	1.5	1.5	6000	0.7	0.6	1.7	0.02	10		
0.3	1.5	1.5	6000	0.7	0.6	1.7	0.02	10	7.598	28.549
0.2	2.5	1.5	6000	0.7	0.6	1.7	0.02	10	12.581	18.622
0.2	1.5	2.5	6000	0.7	0.6	1.7	0.02	10	18.659	55.764
0.2	1.5	1.5	6500	0.7	0.6	1.7	0.02	10	27.461	22.192
0.2	1.5	1.5	6000	0.8	0.6	1.7	0.02	10	6.206	13.556
0.2	1.5	1.5	6000	0.7	0.7	1.7	0.02	10	15.039	21.746
0.2	1.5	1.5	6000	0.7	0.6	1.8	0.02	10	13.132	25.336
0.2	1.5	1.5	6000	0.7	0.6	1.7	0.04	10	15.713	7.406
0.2	1.5	1.5	6000	0.7	0.6	1.7	0.02	15	9.234	26.476

**Table 4:** Variation in the rate of mass transfer grease-based *MoS*_*2*_.

$\phi$	$Nu$	Heat transfer enhancement
0.00	162.42	
0.01	169.783	4.3098%
0.02	177.406	9.2266%
0.03	185.296	14.0844%
0.04	193.466	19.1146%

**Table 5:** Variation in the rate of mass transfer grease-based *MoS*_*2*_.

$\phi$	$Sh$	Decrease in mass distribution
0.00	0.6926	
0.01	0.6967	0.5919%
0.02	0.7010	1.2128%
0.03	0.7054	1.8481%
0.04	0.7100	2.5122%

## Concluding remarks

The goal of this study is to examine closed-form solutions for Maxwell nanofluid flow in open channels. MoS2 nanoparticles are used, while grease is the basis fluid. The Caputo-Fabrizio fractional derivative, which has lately emerged as the most popular fractional derivative, is then used to generalize the classical model. Through the use of the finite Fourier, sine transform and the Laplace transform technique, the coupled system's solutions are achieved. The collected results are also depicted in the graphs. The study's primary outcomes are listed below.

- The variations in all the profiles are shown for different values of α. It is important here to mention that we have different lines for one value of time. This effect is showing the memory effect in the fluid, which cannot be demonstrated from the integer order derivative.
- The present results are reducible to the classical Maxwell nanofluid model by taking $\alpha \to 1$.
- The velocity of Maxwell nanofluid decrease by increasing the amount of MoS_2_ nanoparticles.
- Using nanoparticles in the grease increases the heat transfer rate which will off course increase life time, friction and lubrication in different engines and machinery.
- The Maxwell nanofluid velocity profile increases with respect to $\lambda$, Gm, and $Gr$.
- By rising the value of $\phi$ enhanced heat transfers up to 11.46%.
- The rate of mass transfer decreases to 2.5122% of regular grease.

## Future suggestions

Here are some recommendations for extending the aforementioned challenge for forthcoming researchers.

- Cylindrical co-ordinates can be added to the scope of this issue.
- Various nanoparticles can be added for a variety of purposes.
- The proposed method can be used to represent a variety of non-Newtonian fluids, including second-grade fluid, Jeffery fluid, Couple stress fluid, and others.

## Data Availability

The datasets used and analyzed during the current study available from the corresponding author on reasonable request.
